# Evaluation of different methods for the diagnosis of primary caries lesions: Study protocol for a randomized controlled clinical trial

**DOI:** 10.1371/journal.pone.0273104

**Published:** 2022-08-24

**Authors:** Ana Paula Taboada Sobral, Marcela Letícia Leal Gonçalves, Alexandre Senkiw D´Annibale, Elaine Marcilio Santos, Carolina Cardoso Guedes, Juliana Maria Altavista Sagretti Gallo, Elza Padilha Ferri, Lívia Alves Corrêa Moretti, Lara Jansiski Motta, Alessandro Melo Deana, Anna Carolina Ratto Tempestini Horliana, Sandra Kalil Bussadori

**Affiliations:** 1 School of Dentistry, Universidade Metropolitana de Santos, Santos, Brazil; 2 Postgraduation Program in Health and Environment, Universidade Metropolitana de Santos, Santos, Brazil; 3 Pro-rectory for Academic Affairs, Universidade Metropolitana de Santos, Santos, Brazil; 4 Specialization in Pediatric Dentistry- APCD School of Dentistry, São Paulo, Brazil; 5 Postgraduation Program in Veterinary Medicine in the Coastal Environment, Universidade Metropolitana de Santos, Santos, Brazil; 6 São José dos Campos School of Dentistry, São Paulo State University, São José dos Campos, Brazil; 7 Postgraduation Program in Biophotonics Applied to Health Sciences, Universidade Nove de Julho, São Paulo, Brazil; Obafemi Awolowo University, NIGERIA

## Abstract

**Background:**

According to the World Health Organization (WHO), dental caries is considered one of the greatest pediatric health problems in the world, due to its high prevalence and incidence. Therefore, the early diagnosis of caries lesions is a fundamental procedure for planning treatment aimed at prevention, minimal intervention and promotion of oral health. The present study aims to evaluate, through a randomized and controlled clinical study, which is the best strategy for diagnosing primary caries lesions located in the interproximal region.

**Materials and methods:**

Eighty patients, aged between 4 and 10 years will be randomized and allocated into 2 groups for analysis and comparison of methods for diagnosing caries lesions. The following diagnostic methods will be analyzed and compared: visual clinical examination using ICDAS (International Caries Detection and Assessment System), the iTero Element 5D System (intraoral scanner with near infrared imaging (NIRI) technology) and bitewing radiography (BWX). All evaluations will be carried out by 02 examiners. Examiners will be trained and calibrated to use the visual and radiographic criteria and also to use the iTero 5D intraoral scanner, following the manufacturer’s instructions.

**Trial registration:**

NCT04900246 in ClinicalTrial.gov. First released in 05/11/2021 and last updated in 10/06/2021.

## Introduction

Currently, dental caries is defined as a chronic and infectious disease of multifactorial etiology with a higher incidence in school-age children. According to the World Health Organization (WHO), it is considered one of the greatest pediatric health problems in the world, due to its high prevalence and significant incidence [1, 2].

For years, the main methods used in the dental clinic for diagnosing and evaluating the extent of caries lesions were: visual, tactile and radiographic clinical examination, classified as traditional or conventional detection methods [3]. For radiographic caries detection, the recommended technique is the bitewing (BWX), also called interproximal. When properly performed, it is capable of providing important information to complement the diagnosis, as interproximal radiography allows a better estimate of the more sensitive depth of proximal and occlusal caries in dentin than clinical inspection alone. Furthermore, monitoring of caries lesions can be more reliable and accurate than conventional clinical examination. However, it must be remembered that radiographic images tend to underestimate the real extent of demineralized areas [4–6].

The subjectivity of the visual clinical examination for the diagnosis of caries lesions is a great concern, so, to standardize the diagnoses, indexes can be used as a tool. ICDAS is an acronym for International Caries Detection and Assessment System, that is, an international system for the detection and assessment of caries lesions. This system seeks to standardize the detection of caries lesions and this index can be used for clinical practice, research, teaching and epidemiology [7].

Interproximal radiography helps to identify hard tissue demineralization [8], but a single radiography cannot determine whether demineralization is a sign of active or inactive caries lesions, nor is it able to distinguish between cavitated lesions and lesions with an intact surface [9].

Currently, new technologies have been developed and validated in order to complement and even surpass other methods, such as the Low-Frequency Fluorescent Laser (DIAGNOdent device—Kavo, Germany), the measurement of electrical resistance offered by the dental element (ECM device—LODE, Netherlands), the DIFOTI device, Quantitative Light-Induced Fluorescence (QLF) and Computed Tomography [10, 11].

Near Infrared Imaging (NIRI) technology is a non-ionizing imaging technology that leverages differences in infrared light scattering and absorption depending on the degree of mineralization of the tooth. *In vitro* and *in vivo* studies using NIRI technology for diagnosing caries lesions have yielded encouraging results [12]. Commercially available systems already use this technology to provide grayscale images for diagnosing caries lesions in enamel and dentin [13].

Early diagnosis of caries lesions is a fundamental procedure for planning treatment aimed at prevention, minimal intervention and promotion of oral health. Therefore, this study aims to verify the best strategy for diagnosing caries lesions among visual clinical examination through ICDAS, the iTero Element 5D System (intraoral scanner with NIRI technology) and bitewing radiography (BWX).

## Materials and methods

A randomized, controlled, clinical trial will be conducted. After receiving clarifications regarding the objectives and procedures of the study, the guardians of the children will agree to participate by signing a statement of informed consent.

Eighty individuals who meet the inclusion criteria will be divided into 02 groups (n = 40). For the random distribution of volunteers, randomization will be performed by drawing lots, using the research randomizer program (https://www.randomizer.org/). Group 1: Visual Inspection + BWX Radiographic Assessment + Assessment of the iTero Element 5D scan and Group 2: Visual Inspection + Assessment of the iTero Element 5D scan + BWX Radiographic Evaluation. For each group, the investigator will assess and document the findings of each diagnostic test before moving on to the next test.

### Inclusion criteria

- Patients of any sex who seek dental treatment at the Dentistry Clinic of *Universidade Metropolitana de Santos*.
- Be between 04 and 10 years old;
- Good general health;
- Present at least two tooth surfaces that can be included in the study.

### Exclusion criteria

- Dental surfaces with proximal restorations;
- Surfaces with evident proximal cavities (marginal crest break);
- Absence of adjacent tooth (absence of proximal contact).

### Ethics and dissemination

This study has been approved by the Ethics Committee of *Universidade Metropolitana de Santos* (UNIMES) under process number 4.742.686 and will be conducted in accordance with the norms governing research involving human subjects stipulated in Resolution 466/2012 of the Brazilian National Board of Health. Changes in the protocol will be reported to this same committee.

### Ethics statement

Individuals deemed eligible will receive clarifications regarding the objectives and procedures of the study and those who agree to participate will sign a statement of informed consent. The identity of all individuals will be preserved throughout all stages of the research. No harms are expected.

### Procedures

**Fig 1:**
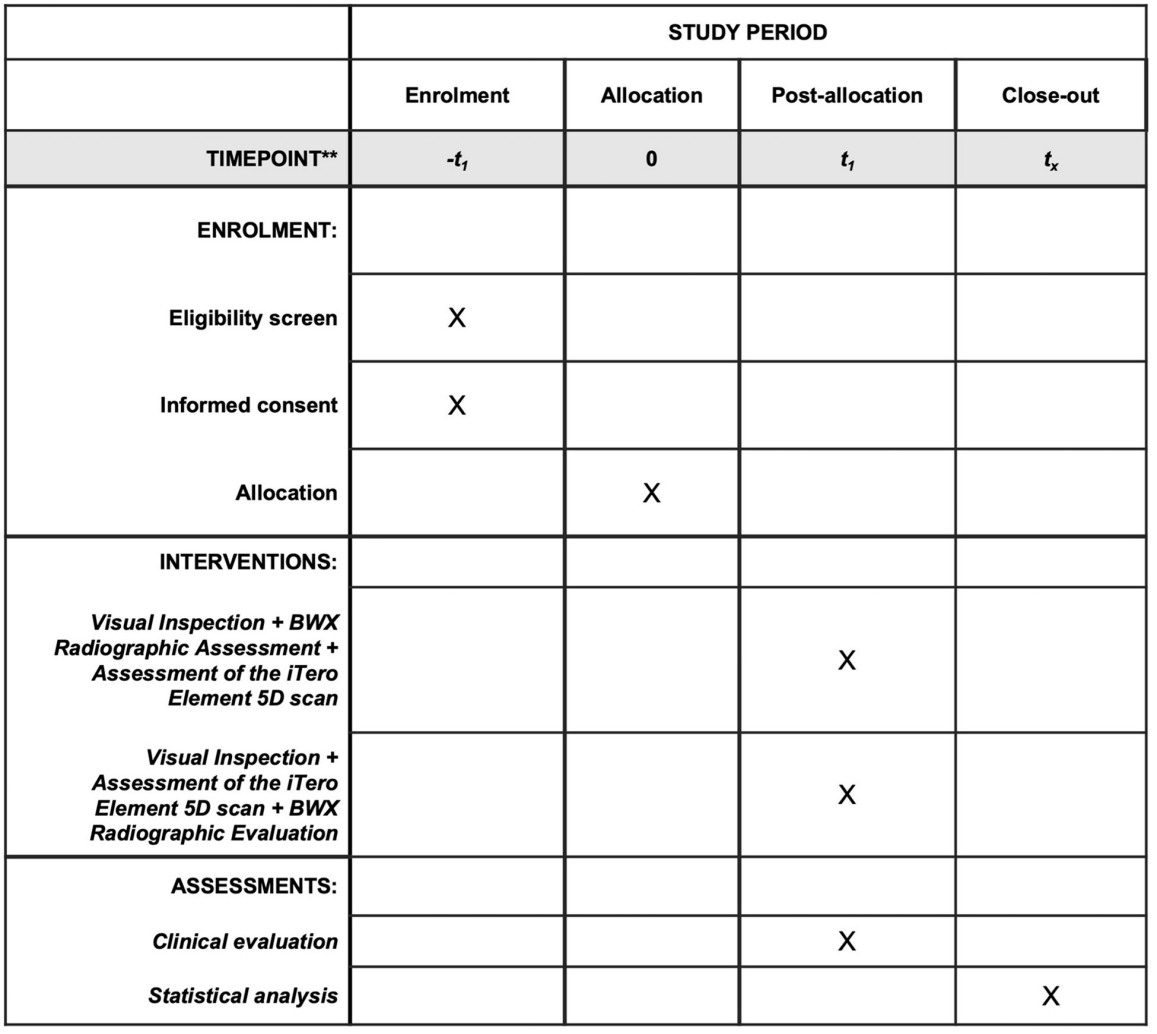
SPIRIT figure as recommended by 2013 SPIRIT statement.

This protocol is in accordance with the 2013 Standard Protocol Items: Recommendations for Interventional Trials (SPIRIT) Statement (Fig 1) and the SPIRIT checklist can be found as S1 File.

### Methods used to diagnose caries lesions

All evaluations will be carried out by 02 examiners. Examiners will be trained and calibrated to use the visual and radiographic criteria and also to use the iTero 5D intraoral scanner, following the manufacturer’s instructions. At the stage of evaluation, according to the different diagnostic strategies tested, the participants will initially receive prophylaxis on their teeth with pumice stone, water and a Robinson brush. On the proximal surfaces, hygiene will be completed with dental floss.

### Visual inspection

The examiners will evaluate the surfaces included in the study independently and without knowledge of the results of the other examiner, after prophylaxis, using a mouth mirror and a WHO probe or "Ball point". The evaluations will be carried out in a dental chair with the aid of a reflector. The teeth will be examined while moist, and afterwards, they will be dried for 5 seconds with the use of a triple syringe.

**Fig 2:**
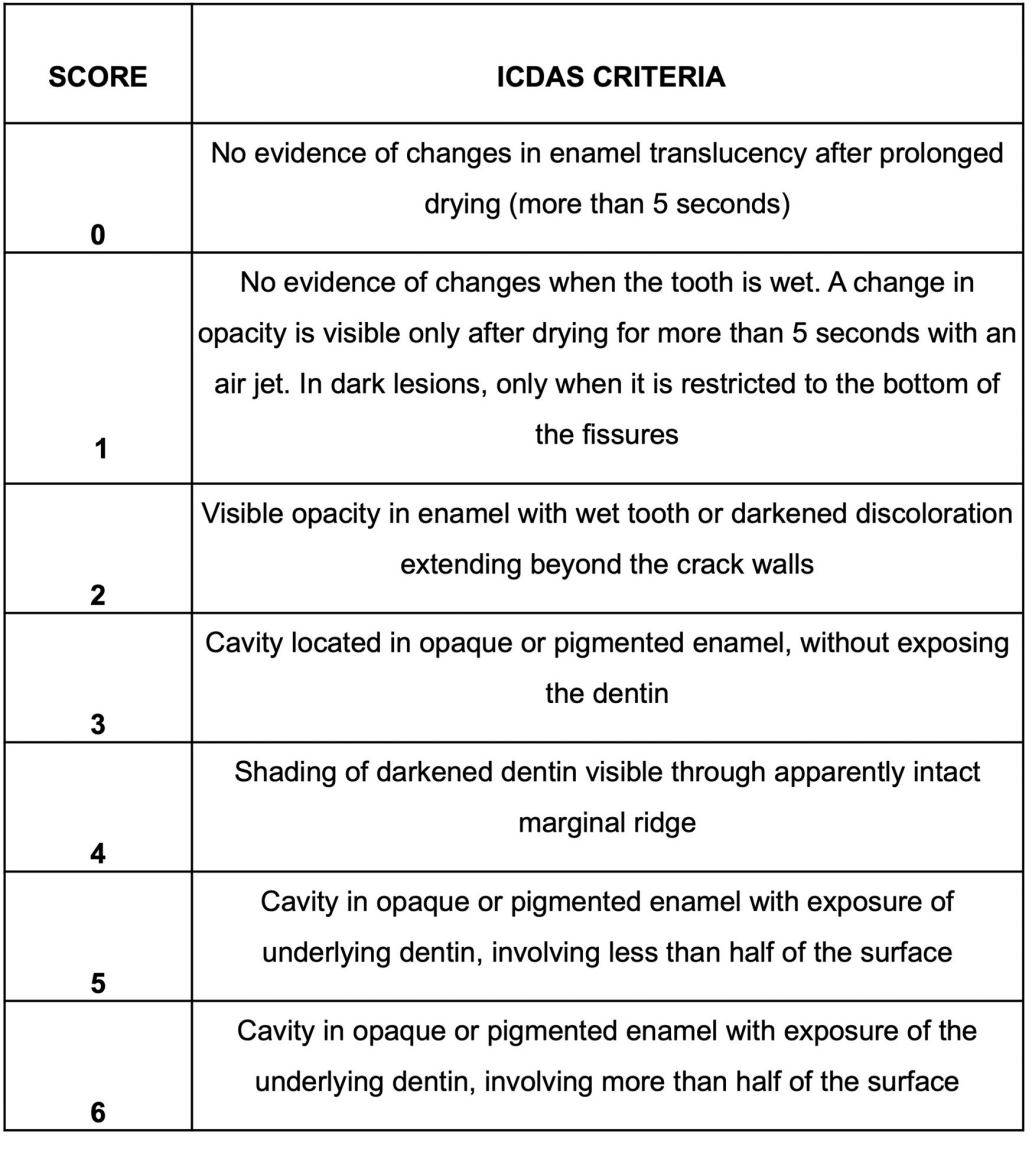
Description of the ICDAS criteria for proximal and occlusal surfaces [14].

For visual inspection, the International Caries Detection and Assessment System (ICDAS) evaluation system will be used. The ICDAS criteria are described in Fig 2.

### BWX radiographic assessment

Participants will be radiographed with a digital X-ray device (DIOX-602, Micro Imagem, Indaiatuba, Brazil) with a regulation of 60 kV and 2 mA and exposure time of 0.10s. The technique used will be the interproximal/bitewing (BWX), with the FIT T1 Digital Sensor (external dimensions: 36.7mm x 24.3mm/sensitive area dimensions: 30.0mm x 20.0mm – 600mm^2^) and the sockets will be standardized with use of Han-Shin radiographic positioners (Jon, São Paulo, Brazil).

### Intra-oral scanning method

For the diagnosis through scanning, the iTero 5D (Align Technology) equipment featuring NIRI technology will be used. This is a diagnostic tool that allows the detection of interproximal and occlusal caries in several stages, from the initial enamel caries to lesions in the amelo-dentin junction, without the use of ionizing radiation. Infrared is the region of the electromagnetic spectrum between 0.7 and 2.0 micrometers (μm). The iTero Element 5D imaging system uses wavelength light (= 850 nm) in an electromagnetic spectrum that, in interaction with the hard tooth tissue, provides additional data on its structure.

### Clinical protocol

The attendance of volunteers will follow the following phases:

1. Anamnesis and Clinical Examination;
2. Verification of eligibility criteria (inclusion and exclusion criteria);
3. Talk with those responsible to inform if the volunteer fits or not the research inclusion criteria. If the volunteer does not fit, he/she/they will be referred to the discipline of Pediatric Dentistry at *Universidade Metropolitana de Santos*;
4. Signature of the Informed Consent Form by the guardians of patients who meet the inclusion criteria. Application form;
5. Visual Inspection (ICDAS);
6. Intraoral Scan: volunteers will be scanned intraorally (iTero Element 5D System). The order of iTero 5D and BWX assessment will be performed according to the study Groups (1 or 2);
7. Radiographic Evaluation: BWX Radiographs;
8. Caries Assessment Form.

The study ends when the volunteers complete all phases of the protocol and follow-up, if necessary. Clinical evaluation and subsequent treatment, if indicated, will be performed upon completion of the study.

### Sample calculation

Sample size can be calculated from the sample size equation for comparing paired nominal data by using McNemar’s test [15]:

$$n=\frac{{\left[z_{\alpha /2}\sqrt{p_{01}+p_{10}}+z_{\beta }\sqrt{p_{01}+p_{10}-{\left(p_{01}-p_{10}\right)}^{2}}\right]}^{2}}{{\left(p_{10}-p_{01}\right)}^{2}}$$

Similar formulae can be obtained for the one-sided non-inferiority test by substituting *z*_*α*/2_, *p*_10_ with *z*_*α*_ and *p*_10_ + *M*:

$$n=\frac{{\left[z_{\alpha }\sqrt{p_{01}+p_{10}+M}+z_{\beta }\sqrt{p_{01}+p_{10}+M-{\left(p_{01}-p_{10}-M\right)}^{2}}\right]}^{2}}{{\left(p_{10}+M-p_{01}\right)}^{2}}$$

where significance level *α* = 0.05, power 1 –β = 0.8, non-inferiority margin M = 5%, assumed detection rate *p*_01_ = 0.06%, *p*_10_ = 0.1%

The minimum sample size required is 12 surfaces. Assuming the drop off rate to be 20%, an additional 25 surfaces will be recruited. So, the total number of surfaces needed for the clinical trial is 149, requiring 80 patients.

### Organization and statistical treatment of data

Non-parametric McNemar’s Chi-Square test will be used for paired nominal data. This test enables the comparison of the detection proportions between the two methods. Kappa coefficients will be calculated to assess the agreement between the two methods.

Differences in detecting the existence of primary interproximal caries lesions above the gingiva (sensitivity and specificity) between the iTero Element 5D system and BWX and the corresponding 90% confidence interval for the differences will be calculated. A minimum of 154 surfaces will ensure a power of 0.8, with an alpha = 0.05. Moderate agreement (Kappa≥0.5) is expected between iTero Element 5D system and radiography. Non-inferiority of the iTero Element 5D is expected as compared with radiography and evaluated by the McNemar’s Chi-Square test.

## Discussion

Despite the wide variety of methods for diagnosing caries lesions, the diagnosis of caries is an extremely fast process, which involves an interpretation of a set of data from clinical signs and complementary tests. Through the present protocol, we will be able to assess whether there will be any difference in effectiveness between the selected diagnostic methods.

## Supporting information

S1 File SPIRIT checklist. (DOC)

S2 File Study protocol in original language. (PDF)

S3 File Statement consent in original language. (PDF)

S4 File Statement consent in English. (PDF)
