# Supplementary material for: Evaluation of different methods for the diagnosis of primary caries lesions: Study protocol for a randomized controlled clinical trial
Source: PLoS One. 2022 Aug 24;17(8):e0273104. doi: 10.1371/journal.pone.0273104 (PMC9401102; doi:10.1371/journal.pone.0273104)
Supplement: S2 File — (PDF) [file pone.0273104.s002.pdf]

Avaliação de diferentes métodos para diagnóstico da lesão de cárie primária: Ensaio clínico randomizado e controlado

Pesquisadora Responsável: Profa. Dra. Ana Paula Taboada Sobral

Santos, 2021

Resumo

De acordo com a Organização Mundial da Saúde (OMS), a cárie dentária é considerada um dos grandes problemas de saúde pediátrica mundial, devido à sua alta prevalência e incidência. Sendo assim, o diagnóstico precoce das lesões de cárie é um procedimento fundamental para a panejamento do plano de tratamento visando a prevenção, a mínima intervenção e a promoção de saúde bucal. O presente estudo tem como objetivo, avaliar por meio de um estudo clínico randomizado e controlado, qual a melhor estratégia para diagnóstico de lesão de cárie primária localizada na região interproximal em indivíduos de 04 a 10 anos. Serão anlisados e comparados os métodos de diagnóstico: exame clínico visual por meio do ICDAS, o Sistema iTero Element 5D (scananer intraoral com tecnologia NIRI) e a radiografia bitewing (BWX). Todas as avaliações serão realizadas por 02 examinadores. Os examinadores serão treinados e calibrados para utilização do critério visual, radiográfico e também para utilização do Scanner intraoral iTero 5D, seguindo a orientação do fabricante.

1. Introdução

Atualmente, a cárie dentária é definida como uma doença crônica e infeciosa de etiologia multifatorial com maior incidência em crianças em idade escolar. De acordo com a Organização Mundial da Saúde (OMS), é considerada como um dos grandes problemas de saúde pediátrica mundial, devido à sua alta prevalência e incidência significativa[1,2].

Durante anos os principais métodos utilizados na clínica odontológica para diagnóstico e avaliação da extensão das lesões de cárie foram: o exame clínico visual, tátil e radiográfico, classificados como métodos tradicionais ou convencionais de detecção[3]. Para detecção radiográfica de cárie, a técnica preconizada é a bitewing,(BWX) chamada também de interproximal. Quando bem realizada, é capaz de fornecer informações importante para complementar o diagnóstico, pois a radiografia interproximal permite uma melhor estimativa da profundidade mais sensível de cáries proximais e oclusais em dentina do que a inspeção clínica isoladamente. Além disso, o monitoramento de lesões de cárie pode ser mais confiável e preciso do que o exame clínico convencional. Porém, é preciso lembrar que as imagens radiográficas tendem a subestimar a real extensão das áreas desmineralizadas [4-6].

A subjetividade do exame clínico visual para o diagnóstico de lesões de cárie é uma grande preocupação, então, para padronizar os diagnósticos podemos utilizar como ferramenta os índices. O ICDAS é um acrônimo de International Caries Detection and Assessment System, ou seja, sistema internacional de detecção e avaliação de lesões de cárie. Esse sistema busca padronizar a detecção de lesões de cárie e o índice pode ser para clínica, pesquisa, ensino e epidemiologia[7].

A radiografia interproximal auxilia na identificação da desmineralização dos tecidos duros[8], mas uma única radiografia não permite determinar se a desmineralização é um sinal de lesão de cárie ativa ou inativa, nem é capaz de distinguir entre lesões cavitadas e lesões com superfície intacta[9].

Na atualidade novas tecnologias e métodos têm sido elaborados e validados com o intuito de complementar e até superar os outros métodos, como o Laser Fluorescente de baixa frequência (aparelho DIAGNOdent - Kavo,Alemanha), a medição da resistência elétrica oferecida pelo elemento dental (aparelho ECM - LODE, Holanda), o aparelho DIFOTI, o Quantitative Light-Induced Fluorescence (QLF) e a Tomografia computadorizada [10,11].

A Tecnologia de Imagem por Infravermelho (NIRI) é uma tecnologia de imagem não ionizante que potencializa as diferenças no espalhamento e na absorção da luz no infravermelho, dependendo do grau de mineralização do dente. Os estudos in vitro e in vivo que usam a tecnologia NIRI para o diagnóstico de lesões de cárie têm produzido resultados encorajadores[12]. Os sistemas disponíveis comercialmente já usam esta tecnologia para fornecer imagens em tons de cinza para diagnóstico de lesões de cárie em esmalte e dentina[13].

O diagnóstico precoce das lesões de cárie é um procedimento fundamental para a panejamento do plano de tratamento visando a prevenção, a mínima intervenção e a promoção de saúde bucal. Sendo assim, o presente estudo tem como proposta, verificar qual é a melhor estratégia para diagnóstico de lesão de cárie, o exame clínico visual por meio do ICDAS, o Sistema iTero Element 5D (scananer intraoral com tecnologia NIRI) e a radiografia bitewing (BWX).

2. Métodos

2.1 Objetivo

O objetivo deste trabalho é avaliar por meio de um estudo clínico randomizado e controlado, qual a melhor estratégia para diagnóstico de lesão de cárie primária localizada na região interproximal; o exame clínico visual (inspeção visual), a tecnologia NIRI e a radiografia bitewing.

2.2 Objetivos Específicos

- Comparar o Sistema iTero Element 5D e a radiografia bitewing para diagnóstico de lesão de cárie primária na região interproximal.
- Comparar o Sistema iTero Element 5D e a radiografia bitewing para diagnóstico de lesão de cárie primária na região oclusal.
- Comparar o Sistema iTero Element 5D e a radiografia bitewing para diagnóstico de trincas e fraturas na região supragengival.

2.3 Delineamento Experimental

O presente trabalho se caracteriza como estudo clínico, randomizado e será realizado nas dependências da Clínica Odontológica da Universidade Metropolitana de Santos (UNIMES). O estudo será registado no site http://www.ensaiosclinicos.gov.br/ de estudos clínicos e será realizado de acordo com as normas do CONSORT (http://www.consort- statement.org/).

2.4 Aspectos Éticos

O estudo será conduzido eticamente de acordo com os critérios descritos na Declaração de Helsinki (World Medical Association Declaration of Helsinki, 2008). O protocolo desse estudo será submetido à aprovação do Comitê de Ética em Pesquisa da Universidade Metropolitana de Santos (UNIMES). Será também informado dos possíveis riscos envolvidos no experimento e da confidencialidade dos dados. Todas as informações estarão presentes no Termo de consentimento livre e esclarecido (Resolução no. 196 do Conselho Nacional de Saúde, Ministério da Saúde, Distrito Federal, Brasil, 10/03/1996), os quais serão assinados em duas vias, pertencendo uma ao voluntário e/ou responsável legal, e outra aos pesquisadores.

Os participantes do estudo receberão também instruções de que poderão desistir do estudo a qualquer momento, por qualquer razão, se assim o desejarem. Os pesquisadores também poderão remover os participantes do estudo, caso achem necessário.

2.5 Determinação do tamanho da amostra

O tamanho da amostra foi calculado a partir da equação do tamanho da amostra para comparar dados nominais emparelhados usando o teste de McNemar[14]:

$$= \frac{[z_{\alpha/2}\sqrt{p_{01}+p_{10}} + z_{\beta}\sqrt{p_{01}+p_{10}-(p_{01}-p_{10})^2}]^2}{(p_{10}-p_{01})^2}$$

Fórmulas semelhantes podem ser obtidas para o teste de não inferioridade unilateral, substituindo por e:$z_{\alpha/2} p_{10} z_{\alpha} p_{10} + M$

$$n = \frac{[z_\alpha\sqrt{p_{01} + p_{10} + M} + z_\beta\sqrt{p_{01} + p_{10} + M - (p_{01} - p_{10} - M)^2}]^2}{(p_{10} + M - p_{01})^2}$$

Nível de significância $\alpha = 0.05$, poder, $1 - \beta = 0.8$ margem de não inferioridade M = 5%, taxa de detecção assumida, $p_{01} = 0.06\%$, $p_{10} = 0.1\%$

O tamanho mínimo da amostra para o presente trabalho é de 12 superfícies. Se a taxa de desistência for de 20%, 25 superfícies adicionais serão necessárias. Portanto, o número total de superfícies para o ensaio clínico é 149. Logo serão necessários 80 pacientes, mas, por margem de segurança visando as possíveis desistências e perdas serão considerados para presente pesquisa 89 pacientes.

2.6 População do Estudo

Na primeira consulta um formulário contendo a história médica do paciente também será preenchido. Na sequência, esses voluntários serão submetidos ao exame clínico, para a determinação das suas condições orais. Com base nas informações coletadas nessa primeira visita, o estudo seguirá os critérios apresentados a seguir:

Após consentimento dos responsáveis e assentimento dos menores, os indivíduos elegíveis foram avaliados para verificar se preenchiam os seguintes critérios de inclusão:

Critérios de Inclusão

- Pacientes de ambos os sexos que buscam tratamento odontológico na Clínica Odontológica da Universidade Metropolitana de Santos.
- Ter entre 04 e 10 anos de idade;
- Apresentar boa saúde geral;
- Apresentar pelo menos duas superfícies passíveis de serem incluídas no estudo.

Critérios de Exclusão

- Superfícies dentárias com restaurações proximais;
- Superfícies com cavidades evidentes proximais (quebra de crista marginal);
- Ausência de dente adjacente (ausência do contato proximal).

2.7 Randomização

Os participantes serão divididos em 02 grupos, conforme descrito na Quadro 1.

Quadro 1. Distribuição dos participantes nos grupos da pesquisa

Grupo	Participantes	Intervenção Terapêutica
1	40	Inspeção Visual + Avaliação Radiográfica BWX + Avaliação do escaneamento iTero Element 5D.
2	40	Inspeção Visual + Avaliação do escaneamento iTero Element 5D.+ Avaliação Radiográfica BWX

Para a distribuição aleatória dos voluntários será realizada a randomização por meio de sorteio, utilizando-se o programa research randomizer (https://www.randomizer.org/)

2.8 Métodos Utilizados para o Diagnóstico de Lesões de Cárie

Todas as avaliações serão realizadas por 02 examinadores. Os examinadores serão treinados e calibrados para utilização do critério visual, radiográfico e também para utilização do Scanner intraoral iTero 5D, seguindo a orientação do fabricante.

Nesta etapa de avaliação segundo as diferentes estratégias de diagnóstico testadas, os participantes inicialmente receberam profilaxia nos dentes com pedra pomes, água e escova de Robinson. Nas superfícies proximais, a higiene foi concluída utilizando o fio dental.

2.8.1 Inspeção Visual

Os examinadores avaliarão as superfícies incluídas no estudo de forma independente e sem conhecimento dos resultados do outro examinador, após profilaxia, utilizando espelho bucal e sonda OMS ou “Ball point”. As avaliações serão realizadas em cadeira odontológica com auxílio de refletor. Os dentes serão examinados úmidos, e posteriormente, será realizada a secagem por 5 segundos com o uso da seringa tríplice.

Para Inspeção Visual utilizaremos o sistema de avaliação ICDAS. O critério ICDAS está descrito no Quadro 2.

Quadro 2. Descrição do critério ICDAS para superfícies proximais e oclusais[14] (Ismail etal., 2007)

ESCORE	CRITÉRIO ICDAS
0	Nenhuma evidência de alterações na translucidez do esmalte após secagem prolongada (mais que 5 segundos)
1	Nenhuma evidência de alterações quando o dente é visualizado úmido, somente após secagem maior que 5 segundos com jato de ar, uma mudança na opacidade é visível. Emlesões escuras, apenas quando é restrita ao fundo das fissuras
2	Opacidade visível no esmalte com o dente úmido ou descoloração escurecida ultrapassando as paredes da fissura
3	Cavidade localizada em esmalte opaco oupigmentado, sem expor a dentina
4	Sombreamento da dentina escurecida visívelatravés da crista marginal aparentemente intacta
5	Cavidade em esmalte opaco ou pigmentadocom exposição da dentina subjacente envolvendo menos da metade da superfície
6	Cavidade em esmalte opaco ou pigmentadocom exposição da dentina subjacente, envolvendo mais da metade da superfície

2.8.2 Método Radiográfico

Os participantes serão radiografados com aparelho de raios X convencional (Spectro 70X, Dabi Atlante, Riberão Preto, Brasil) com regulagem de 70 kV e 8 mA e tempo de exposição de 0,8. A técnica utilizada será a interproximal /bitewing

(BWX), com filmes radiográficos periapicais 31 X 41 mm e as tomadas serão padronizadas com uso de posicionadores radiográficos Han-Shin (Jon, São Paulo, Brasil). Na etapa de revelação manual, a padronização será realizada pelo método tempo-temperatura.

2.8.3 Método Scaneamento Intra-Oral

Para o scaneamento será utilizado o equipamento iTero 5D (Align Technology) que apresenta a Tecnologia NIRI. A Tecnologia de Imagem por Infravermelho (NIRI) é uma ferramenta de diagnostico que permite a detecção de cárie interproximal e oclusal em vários estágios, desde a cárie inicial no esmalte até lesões na junção amelo-dentinária, sem a utilização de radiação ionizante. O Infravermelho é a região do espectro eletromagnético entre 0,7 e 2,0 micrômetros (µm). O sistema de imagem iTero Element 5D utiliza luz de comprimento de onda (= 850 nm) em um espectro eletromagnético que, em interação com o tecido duro do dente, fornece dados adicionais de sua estrutura.

2.8.4 Protocolo de Atendimento

O atendimento dos voluntários seguirá as seguintes fases:

1. Anamnese e Exame Clínico
2. Verificação dos critérios de elegibilidade (critérios de inclusão e exclusão)
3. Conversa com os responsáveis para informar se o voluntário se enquadra ou não nos critérios de inclusão da pesquisa. Caso o voluntário não se enquadre, o mesmo será encaminhado para atendimento na disciplina de Odontopediatria da Universidade Metropolitana de Santos.
4. Assinatura do Termo de Consentimento Livre e Esclarecido pelos responsáveis dos pacientes que se enquadram nos critérios de inclusão. Formulário de inscrição
5. Inspeção Visual (ICDAS)
6. Escaneaneto intraoral: os voluntários serão escaneados intraoralmente (Sistema iTero Element 5D). A ordem de avaliação iTero 5D e BWX será realizada de acordo com o grupo do paciente.
7. Avaliação Radiográfica: Radiografias BWX

8. Preenchimento do Formulário de Avaliação de Cárie

O término do estudo ocorrerá quando os voluntários concluírem todas as fases do protocolo e de acompanhamento, se necessário. A avaliação clínica e o tratamento subsequentes, se indicados, serão realizados após a conclusão do estudo.

2.9 Forma de análise dos resultados

Todos as análises serão realizadas de forma separada para os diferentes métodos utilizados para o diagnóstico de lesões de cárie. Inicialmente, serão calculados os valores relacionados à concordância inter e intraexaminador obtidos com os métodos realizados de forma isolada.

2.9.1 Análise Estatística

Serão calculadas as diferenças na detecção da existência de lesões de cárie primária na região interproximal (sensibilidade e especificidade) entre o sistema iTero Element 5D e BWX e o intervalo de confiança de 90% correspondente para as diferenças. Um mínimo de 154 superfícies garantirá uma potência de 0,8, com um alfa = 0,05.

Espera-se uma concordância moderada (Kappa≥0,5) entre o sistema iTero Element 5D e a radiografia. A não inferioridade do iTero Element 5D é esperada em comparação com a radiografia e avaliada pelo teste Qui-quadrado de McNemar.

3. Resultados Esperados

Apesar da grande variedade dos métodos para o diagnóstico das lesões de cárie, o diagnóstico da doença cárie é um processo extremamente complexo, que envolve a interpretação de um conjunto de dados provenientes dos sinais e sintomas clínicos e de exames complementares. Por meio do presente trabalho poderemos avaliar, se haverá diferença de efetividade entre os entre os métodos de diagnóstico avaliados.

4. Cronograma de Execução

**Atividades**	Pré intervenção			1	2	3	4	5	6
Submissão Comitê de Ética em Pesquisa									
Seleção dos participantes e randomização									
Procedimentos									
Coleta dos dados									
Análise dos dados									
Redação e apresentação dos resultados obtidos									
Submissão dos artigos científicos									
Relatório final									

5. Referencias

1. Angelopoulou M V., Beinlich M, Crain A. Early Childhood Caries and Weight Status: A Systematic Review and Meta-Analysis. Pediatr Dent. 2019;41(4):261-72.

2. World Health Organization. Ending childhood dental caries. 2019

3. Leão Filho, Jorge César Borges, and Thayse Rodrigues de Souza. "Métodos de detecção de cárie: do tradicional às novas tecnologias de emprego clínico." *Revista de Odontologia da Universidade Cidade de São Paulo* 23.3 (2017): 253-265.

4. Braga, M. M., Mendes, F. M., Ekstrand, K. R. Detection activity assessment and diagnosis of dental caries lesions. Dent. Clin. North. Am. 2010; 54 (3): 479-93.

5. Purger, F. P., Oliveira, P. R. A., Vasconcellos, A. et al. Relative importance of radiographs in diagnosing primary molar´s proximal caries. Journal Dental Re

6. Wenzel, A. Bitewing and digital bitewing radiography for detection of caries lesions. J. Dent. Res. 2004; 83 (Spec No C): C72-5.

7. Braga, Ma Minatel et al. O uso do ICDAS para diagnóstico e planejamento do tratamento da doença cárie. PRO-odonto prevenção, v. 5, n. 4, p. 9-55, 2012.

8. Wenzel A, Hirsch E, Christensen J, Matzen LH, Scaf G, Frydenberg M. Detection of cavitated approximal surfaces using cone beam CT and intraoral receptors. Dentomaxillofac Radiol 2013;42:39458105.

9. Mariath AAS, Casagrande L, de Araujo FB. Grey levels and radiolucent lesion depth as cavity predictors for approximal dentin caries lesions in primary teeth. Caries Res 2007;36:377–81.

10. Mialhe FL, Bosquiroli V, Silva JO. Conhecimento e utilização de métodos de detecção de lesões cariosas por cirurgiões-dentistas. Varia Scientia 2005 5(10):23-33

11. Murdoch-Kinch CA, McLean ME. Minimally invasive dentistry. J Am Dent Assoc 2003 Jan;134(1):87-95.

12. Fried et al. 2005; Simon, Darling et al. 2016; Simon, Lucas et al. 2016; Litzenburger et al. 2018

13.Söchtig et al. 2014; Abdelaziz e Krejci 2015; Kühnisch et al. 2016; Abdelaziz et al. 2018.

14. Kühnisch J. In vivo validation of near-infrared light transillumination for interproximal dentin caries detection. Clin Oral Investig. 2016 May;20(4):821-9.
