# Supplementary material for: Evaluation of different methods for the diagnosis of primary caries lesions: Study protocol for a randomized controlled clinical trial
Source: PLoS One. 2022 Aug 24;17(8):e0273104. doi: 10.1371/journal.pone.0273104 (PMC9401102; doi:10.1371/journal.pone.0273104)
Supplement: S3 File — (PDF) [file pone.0273104.s003.pdf]

UNIVERSIDADE METROPOLITANA DE SANTOS - UNIMES

COMITÊ DE ÉTICA EM PESQUISA

TERMO DE CONSENTIMENTO LIVRE E ESCLARECIDO

I - DADOS DE IDENTIFICAÇÃO DO SUJEITO DA PESQUISA OU RESPONSÁVEL LEGAL

1. NOME DO PACIENTE:
DOCUMENTO DE IDENTIDADE Nº: SEXO : M ( ) F ( )
DATA NASCIMENTO (dd/mm/aaaa):
ENDEREÇO Nº APTO:
BAIRRO: CIDADE
CEP: TELEFONE: DDD ( )

2.RESPONSÁVEL LEGAL
NATUREZA (grau de parentesco, tutor, curador etc.)
DOCUMENTO DE IDENTIDADE: SEXO: M ( ) F ( )
DATA NASCIMENTO.:
ENDEREÇO: Nº Comp.:
BAIRRO: CIDADE:
CEP: TELEFONE: DDD ( )

II - DADOS SOBRE A PESQUISA CIENTÍFICA

1. TÍTULO DO PROTOCOLO DE PESQUISA: Avaliação de diferentes métodos para diagnóstico da lesão de cárie primária: Ensaio clínico randomizado e controlado

2. PESQUISADOR: Ana Paula Taboada Sobral

CARGO/FUNÇÃO: Professora do Curso de Odontologia da UNIMES

INSCRIÇÃO CONSELHO REGIONAL Nº 76.693

UNIDADE DA UNIMES: **Faculdade de Odontologia -** Av. Conselheiro Nébias, 536 - Encruzilhada, Santos - SP, 11045-002

3. AVALIAÇÃO DO RISCO DA PESQUISA:

SEM RISCO ( ) RISCO MÍNIMO (x) RISCO MÉDIO ( )

RISCO BAIXO ( ) RISCO MAIOR ( )

(probabilidade de que o indivíduo sofra algum dano como consequência imediata ou tardia do estudo)

4.DURAÇÃO DA PESQUISA: 06 meses

III - REGISTRO DAS EXPLICAÇÕES DO PESQUISADOR AO PACIENTE OU SEU REPRESENTANTE LEGAL SOBRE A PESQUISA CONSIGNANDO:

1. **Justificativa e os objetivos da pesquisa:**

O diagnóstico precoce das lesões de cárie é um procedimento fundamental para a panejamento do plano de tratamento visando a prevenção, a mínima intervenção e a promoção de saúde bucal. Sendo assim, o presente estudo tem como proposta, verificar qual é a melhor estratégia para diagnóstico de lesão de cárie, o exame clínico visual por meio do ICDAS, o Sistema iTero Element 5D (scananer intraoral com tecnologia NIRI) e a radiografia bitewing (BWX).

2. **Procedimentos que serão utilizados e propósitos, incluindo a identificação dos procedimentos que são experimentais:**

Todas as avaliações serão realizadas por 02 examinadore. Os examinadores serão treinados e calibrados para utilização do critério visual, radiográfico e também para utilização do Scanner intraoral iTero 5D, seguindo a orientação do fabricante.

Nesta etapa de avaliação segundo as diferentes estratégias de diagnóstico testadas, os participantes inicialmente receberam profilaxia nos dentes com pedra pomes, água e escova de Robinson. Nas superfícies proximais, a higiene foi concluída utilizando o fio dental.

Os participantes serão divididos em dois Grupos:

Grupo 1. Inspeção Visual + Avaliação Radiográfica BWX + Avaliação do escaneamento iTero Element 5D.

Grupo 2. Inspeção Visual + Avaliação do escaneamento iTero Element 5D + Avaliação Radiográfica BWX .

O paciente virá no máximo a 4 (quatro) consultas com o tempo de 30 minutos cada.

3. **Desconfortos e riscos esperados:**
   Desconforto durante a realização do exame rádiografico e escaneamento.
   a. Os exames clínicos e radiográficos farão parte do prontuário do atendimento da faculdade.
   b. O escaneamento com iTero Element 5D não causa nenhum dano à saúde caso seja usado de acordo com as normas do fabricante.
   c. O número de radiografias a serem tomadas neste estudo é muito menor do que a quantidade que pode causar risco à saúde da pessoa.

4. **Benefícios que poderão ser obtidos:**
   Os voluntários e seus responsáveis participarão das atividades de educação em saúde bucal com aconselhamento de alimentação e higiene.
   Os voluntários terão a boca examinada e se houver necessidade serão encaminhadas para tratamento odontológico.

5. **Procedimentos alternativos que possam ser vantajosos para o indivíduo:** Não serão utilizados métodos alternativos.

IV - ESCLARECIMENTOS DADOS PELO PESQUISADOR SOBRE GARANTIAS DO SUJEITO DA PESQUISA CONSIGNANDO:

1. Acesso, a qualquer tempo, às informações sobre procedimentos, riscos e benefícios relacionados à pesquisa, inclusive para dirimir eventuais dúvidas.

2. Liberdade de retirar seu consentimento a qualquer momento e de deixar de participar do estudo, sem que isto traga prejuízo à continuidade da assistência.

3. Salvaguarda da confidencialidade, sigilo e privacidade.

4. Disponibilidade de assistência, por eventuais danos à saúde, decorrentes da pesquisa.

5. Vabilidade de indenização por eventuais danos à saúde decorrentes da pesquisa.

V. INFORMAÇÕES DE NOMES, ENDEREÇOS E TELEFONES DOS RESPONSÁVEIS PELO ACOMPANHAMENTO DA PESQUISA, PARA CONTATO EM CASO DE INTERCORRÊNCIAS CLÍNICAS E REAÇÕES ADVERSAS.

Profa. Dra. Ana Paula Taboada Sobral

Contatos: (11) 98447-4570 / anapaula@taboada.com.br

Faculdade Odontologia UNIMES - Av. Conselheiro Nébias, 536 - Encruzilhada, Santos – SP-CEP: 11045-002

VI. OBSERVAÇÕES COMPLEMENTARES:

Não se aplica.

VII - CONSENTIMENTO PÓS-ESCLARECIDO

Declaro que, após convenientemente esclarecido pelo pesquisador e ter entendido o que me foi explicado, consinto em participar do presente Protocolo de Pesquisa

Santos, de de 20 .

______________________________ ______________________________

Assinatura do Responsável Legal

Assinatura do pesquisador
(carimbo ou nome Legível)
