# Supplementary material for: Evaluation of different methods for the diagnosis of primary caries lesions: Study protocol for a randomized controlled clinical trial
Source: PLoS One. 2022 Aug 24;17(8):e0273104. doi: 10.1371/journal.pone.0273104 (PMC9401102; doi:10.1371/journal.pone.0273104)
Supplement: S4 File — (PDF) [file pone.0273104.s004.pdf]

**METROPOLITAN UNIVERSITY OF SANTOS - UNIMES**

**RESEARCH ETHICS COMMITTEE**

# FREE AND INFORMED CONSENT FORM

---

## I - IDENTIFICATION DATA OF THE RESEARCH SUBJECT OR LEGAL PERSON

**1**. PATIENT NAME:
IDENTITY DOCUMENT No.: GENDER: M ( ) F ( )
DATE OF BIRTH (dd/mm/yyyy):
ADDRESS
STATE: CITY
Zip code: PHONE:

**2.**LEGAL RESPONSIBLE
NATURE (degree of kinship, tutor, curator, etc.)
IDENTITY DOCUMENT: GENDER: M ( ) F ( )
BIRTH DATE.:
ADDRESS: Comp No.:
STATE: CITY
Zip code: PHONE:

---

## II - DATA ON SCIENTIFIC RESEARCH

**1.** TITLE OF THE RESEARCH PROTOCOL: Evaluation of different methods for diagnosing primary caries lesion: Randomized and controlled clinical trial

**2.** RESEARCHER: Ana Paula Taboada Sobral

POSITION/FUNCTION: Professor of the Dentistry Course at UNIMES

REGIONAL COUNCIL REGISTRATION No. 76,693

UNIT OF UNIMES: **Faculty of Dentistry -** Av. Conselheiro Nébias, 536 - Encruzilhada,
Santos - SP, 11045-002

**3**. RESEARCH RISK ASSESSMENT:

NO RISK( ). MINIMUM RISK (x) MEDIUM RISK( )

LOW RISK ( ) HIGHER RISK ( )

(probability that the individual will suffer some damage as an immediate or late consequence of the study)

**4**. SURVEY DURATION: 06 months

## III - RECORD OF THE RESEARCHER'S EXPLANATIONS TO THE PATIENT OR THEIR LEGAL REPRESENTATIVE ABOUT THE RESEARCH CONSIDERING:

1. **Rationale and research objectives:**

Early diagnosis of caries lesions is a fundamental procedure for planning the treatment plan aimed at prevention, minimal intervention, and promotion of oral health. Therefore, the present study aims to verify which is the best strategy for diagnosing caries lesions, visual clinical examination through ICDAS, the iTero Element 5D System (intraoral scanner with NIRI technology) and bitewing radiography (BWX ).

2. **Procedures that will be used and purposes, including identification of procedures that are experimental:**

All assessments will be carried out by 02 examiners. Examiners will be trained and calibrated to use visual and radiographic criteria and to use the iTero 5D intraoral scanner, following the manufacturer's instructions.

At this stage of assessment, according to the different diagnostic strategies tested, the participants initially received prophylaxis on their teeth with pumice stone, water, and a Robinson brush. On the proximal surfaces, hygiene was completed using dental floss.

Participants will be divided into two groups:

Group 1. Visual Inspection + BWX Radiographic Evaluation + Evaluation of the iTero Element 5D scan.

Group 2. Visual Inspection + Evaluation of the iTero Element 5D scan + BWX Radiographic Evaluation.

The patient will come to a maximum of 4 (four) appointments with a time of 30 minutes each.

3. **Desconfortos and expecte risks:**
   Discomfort during radiographic examination and scanning.
   The. Clinical and radiographic examinations will be part of the faculty's medical record.
   B. Scanning with iTero Element 5D does not cause any harm to health if used in accordance with the manufacturer's regulations.
   ç. The number of radiographs to be taken in this study is much less than the amount that could pose a risk to the person's health.

4. **Benefits that can be obtained:**
   Volunteers and their guardians will participate in oral health education activities with food and hygiene counseling. The volunteers will have their mouth examined and if necessary, they will be referred for dental treatment.

5. **Alternative procedures that may be beneficial to the individual:** Alternative methods will not be used.

---

## IV - CLARIFICATIONS GIVEN BY THE RESEARCHER ABOUT GUARANTEES OF THE SUBJECT OF THE RESEARCH CONSIDERING:

**1**. Access, at any time, to information about procedures, risks and benefits related to the research, including to resolve any doubts.

**two**. Freedom to withdraw your consent at any time and to stop participating in the study, without prejudice to the continuity of care.

**3**. Safeguarding confidentiality, secrecy, and privacy.

**4**. Availability of assistance, for possible damage to health resulting from the research.

**5**. Possibility of indemnity for any damage to health resulting from the research.

---

**V. INFORMATION ON NAMES, ADDRESSES AND PHONE PHONES OF THOSE RESPONSIBLE FOR MONITORING THE RESEARCH, FOR CONTACT IN CASE OF CLINICAL INTERCORRENCES AND ADVERSE REACTIONS.**

Prof. Dra. Ana Paula Taboada Sobral

Contacts: (11) 98447-4570 / anapaula@taboada.com.br

**UNIMES Dentistry Faculty -** Av. Conselheiro Nébias, 536 - Encruzilhada, Santos – SP-CEP: 11045-002

---

**SAW. ADDITIONAL NOTES**:

---

Not applicable.

---

**VII - POST-INFORMED CONSENT**

I declare that, after being conveniently clarified by the researcher and having understood what was explained to me, I consent to participate in this Research Protocol

Sntos, / /. .

______________________________________ ______________________________________

Signature of Legal Responsible Signature of Researcher
(Stamp or legible name)
